# SHP2 Nuclear/Cytoplasmic Trafficking in Granulosa Cells Is Essential for Oocyte Meiotic Resumption and Maturation

**DOI:** 10.3389/fcell.2020.611503

**Published:** 2021-01-22

**Authors:** Muhammad Idrees, Vikas Kumar, Myeong-Don Joo, Niaz Ali, Keun-Woo Lee, Il-Keun Kong

**Affiliations:** ^1^Division of Applied Life Science (BK21 Four), Institute of Agriculture and Life Science (IALS), Gyeongsang National University, Jinju, South Korea; ^2^Division of Applied Life Science, Department of Bio and Medical Big Data (BK21 Four), Research Institute of Natural Science (RINS), Gyeongsang National University (GNU), Jinju, South Korea; ^3^Institute of Basic Medical Sciences, Khybar Medical University, Peshawar, Pakistan; ^4^The King Kong Corp. Ltd., Gyeongsang National University, Jinju, South Korea

**Keywords:** granulosa cells, SHP2, ER-α, Nppc/Npr2, ERK1/2, COV434 cell line, protein-protein docking, molecular dynamics simulations

## Abstract

Src-homology-2-containing phosphotyrosine phosphatase (SHP2), a classic cytoplasmic protein and a major regulator of receptor tyrosine kinases and G protein-coupled receptors, plays a significant role in preimplantation embryo development. In this study, we deciphered the role of SHP2 in the somatic compartment of oocytes during meiotic maturation. SHP2 showed nuclear/cytoplasmic localization in bovine cumulus and human granulosa (COV434) cells. Follicle-stimulating hormone (FSH) treatment significantly enhanced cytoplasmic SHP2 localization, in contrast to the E_2_ treatment, which augmented nuclear localization. Enhanced cytoplasmic SHP2 was found to negatively regulate the expression of the ERα-transcribed *NPPC* and *NPR2* mRNAs, which are vital for oocyte meiotic arrest. The co-immunoprecipitation results revealed the presence of the SHP2/ERα complex in the germinal vesicle-stage cumulus–oocyte complexes, and this complex significantly decreased with the progression of meiotic maturation. The complex formation between ERα and SHP2 was also confirmed by using a series of computational modeling methods. To verify the correlation between SHP2 and *NPPC*/*NPR2*, SHP2 was knocked down *via* RNA interference, and *NPPC* and *NPR2* mRNAs were analyzed in the control, E_2_, and FSH-stimulated COV434 cells. Furthermore, phenyl hydrazonopyrazolone sulfonate 1, a site-directed inhibitor of active SHP2, showed no significant effect on the ERα-transcribed *NPPC* and *NPR2* mRNAs. Taken together, these findings support a novel nuclear/cytoplasmic role of SHP2 in oocyte meiotic resumption and maturation.

## Introduction

In mammals, dormant oocytes surrounded by somatic cells enter meiotic cell division during embryonic development and meiotic arrest during the first meiotic prophase (MI) stage for a prolonged period (Guo et al., 2018). The communication between the oocyte and its surrounding somatic cells (including theca, mural granulosa, and cumulus cells) is critical for maintaining this stage, as meiosis inhibitory signals pass from somatic cells to oocytes *via* gap junctions. Several molecular mechanisms in somatic cells work together to preclude the activation of meiotic-progression-related machinery in oocytes (Zhang et al., 2010). The estradiol (E_2_)–estrogen receptor (ER) system is one of the mechanisms of oocyte meiotic arrest maintained by granulosa cells (Liu et al., 2017). E_2_, a steroid hormone, is a critical regulator of oocyte meiotic arrest and exerts its effects by binding to ERα and ERβ (Lubahn et al., 1993). Ligand-activated ERα regulates the transcription of natriuretic peptide type C (*NPPC*) and natriuretic peptide receptor 2 (*NPR2*) by directly binding to their promoter regions and enhancing their expression in granulosa cells (Liu et al., 2017). The *NPPC* and *NPR2* genes further regulate the production of cyclic guanosine monophosphate in granulosa cells, which then enters the oocyte *via* gap junctions, inhibit phosphodiesterase 3A-induced cyclic adenosine monophosphate degradation, and maintain oocyte meiotic arrest (Zhang et al., 2010; Kiyosu et al., 2012; Wigglesworth et al., 2013).

Follicle-stimulating hormone (FSH), a negative feedback regulator of ERα, controls folliculogenesis by stimulating the granulosa cells of early antral follicles (Glidewell-Kenney et al., 2008; El-Hayek et al., 2014). FSH transduces its signals *via* the FSH-specific G protein-coupled receptor (FSHR), depending on intracellular effectors (Casarini and Crepieux, 2019). By activating mitogen-activated protein kinase (MAPK)/extracellular signal-regulated kinase (ERK) and AKT (protein kinase B) signaling pathways, FSH induces the proliferation and differentiation of granulosa cells to a pre-ovulatory phenotype (El-Hayek et al., 2014; Donaubauer et al., 2016). FSH requires several proteins to transduce its signaling in granulosa cells, and SHP2 has been identified to play a key role in FSH signaling (Donaubauer et al., 2016). SHP2, a main regulating component of receptor tyrosine kinases (RTKs) and G protein coupled receptors (GPCRs), shows ubiquitous expression and has multiple functions (Qu, 2002; Zhang et al., 2004; Furcht et al., 2014). Evidence has shown that SHP2 is critical for numerous reproductive tissues (Hu et al., 2015, 2017; Ran et al., 2017; Idrees et al., 2019b, 2020; Kim et al., 2019). By interacting with the transmembrane adaptor proteins, SHP2 induces the sustained activation of the rat sarcoma (RAS) and ERK1/2 signaling pathway, which is essential for oocyte meiotic maturation and ovulation (Fan and Sun, 2004; Dance et al., 2008; Fan et al., 2009). In addition to folliculogenesis, SHP2 is important for embryonic development, as indicated by SHP2-knockout embryos which exhibit inner cell mass death and failure to yield trophoblast stem cells (Saxton et al., 1997; Yang et al., 2006).

The primary function of SHP2 is to positively affect the intracellular signaling of RTKs and GPCRs in the cytoplasm, but unconventional nuclear localization of SHP2 has also been identified by several studies (Jakob et al., 2008; Li et al., 2014; Ran et al., 2017). The nucleus-localized SHP2 is essential for the activity of several nuclear proteins, such as nuclear SHP2, which retain telomerase reverse transcriptase in the nucleus for telomere lengthening (Jakob et al., 2008). SHP2 is required for signal transducer and activator of transcription 5 nuclear translocation and transcriptional activity (Chughtai et al., 2002). ERα, an essential protein for fertility, also requires nuclear SHP2 for DNA binding, as SHP2 enhances the Src kinase-mediated tyrosine phosphorylation of ERα in the uterus and facilitates its binding to the progesterone promoter region (Ran et al., 2017). Cytoplasmic SHP2 and extra-nuclear ERα also form a complex and significantly affect mitogen-activated protein (MAP) kinases and AKT signaling (Levin, 2009; Li et al., 2014).

In this study, we identified a novel role of SHP2 in association with ERα and FSH in mammalian oocyte meiotic resumption. Our results provide an experimental proof that SHP2 exists in complex with ERα in the nucleus of human granulosa and bovine cumulus cells. Progression in bovine oocyte meiotic maturation significantly reduced the SHP2/ERα complex. The FSH-mediated stimulation of cultured granulosa cells also reduced the SHP2/ERα complex as compared to E_2_ treatment that markedly enhanced it. Furthermore, FSH increases the cytoplasmic localization of SHP2, and this may be due to FSHR-induced signaling in granulosa cells. Moreover, the E_2_ activation of ERα not only enhanced the mRNA expression of *NPPC* and *NPR2* but also increased the nuclear localization of SHP2. The functional role of SHP2 in oocyte-surrounding somatic cells and its significance in oocyte maturation have never been studied. Thus, the elucidation of the SHP2 mechanism could provide a potential therapeutic target in the treatment of infertility.

## Materials and Methods

The study was conducted in full compliance with the Gyeongsang National University Institute of Animal Care Committee (GNU-130902-A0059). Most of the chemicals and reagents were obtained from Sigma-Aldrich (St. Louis, MO, USA), unless otherwise noted.

### Experiment Procedures

#### Experiment 1

To understand the role of SHP2 in the somatic compartment of oocytes, bovine ovary-derived cumulus–oocyte complexes (COCs) were used to culture cumulus cells. Primary bovine cumulus cells were separated from pre-ovulatory follicles and cultured in α-MEM media. FSH (20 ng/ml) and epidermal growth factor (EGF 25 ng/ml) were used to stimulate the cultured cumulus cells. Phenyl hydrazonopyrazolone sulfonate 1 (PHPS1) (5 μM) was used for COCs and cultured cumulus cells according to our previous study (Idrees et al., 2019b). Samples from various stages of bovine oocytes were used to examine the SHP2/ERα complex *via* co-immunoprecipitation (co-IP) and immunofluorescence.

#### Experiment 2

To analyze the SHP2/ERα complex in granulosa cells, COV434 human granulosa cell line was used, and co-IP was used to detect complex formation. 3-(4,5-Dimethylthiazol-2-yl)-2,5-diphenyltetrazolium bromide (MTT) assay showed that the least effective concentration of PHPS1 in the COV434 cell line was 3 μM. For FSH and E_2_, the same effective concentrations were used as those in a previous study that used the COV434 cell line (Liu et al., 2017). SHP2 was knocked down using small interfering RNA (siRNA, 10 nM). The expression of ERα was examined *via* immunofluorescence, and its transcribed genes (*NPPC* and *NPR2*) were analyzed *via* quantitative real-time polymerase chain reaction (RT-qPCR).

### COC Collection and *in vitro* Maturation

Local abattoir-derived bovine ovaries were transported to the laboratory within 2 h after dissection. COCs with follicle diameter ranging from 3 to 6 mm and with a minimum of three layers of cumulus cells were collected according to a previously described protocol (Idrees et al., 2019b). In brief, ovaries were washed with Dulbecco's phosphate-buffered saline (D-PBS), and COCs were aspirated using 18-gauge disposable needles attached to a vacuum pump. The follicular medium was diluted using TL-HEPES [10 mM HEPES (H-6147), 2 mM calcium chloride (C-7902), 3.2 mM potassium chloride (P-5405), 0.5 mM magnesium chloride (M-2393), 10 mM sodium lactate, 2 mM sodium bicarbonate (S-5761), 114 mM sodium chloride (S-5886), 0.34 mM sodium bisphosphate (S-5011), 1 μl/ml phenol red, 100 IU/ml penicillin, and 0.1 mg/ml streptomycin]. A stereomicroscope was used to collect COCs with a minimum of three uniform layers. The retrieved GV-stage oocytes, along with cumulus cells in groups of 40–50, were cultured in 700 μl of TCM199 media (Invitrogen Corp., Carlsbad, CA, USA) supplemented with 10% (v/v) fetal bovine serum (FBS; Gibco BRL, Life Technologies, Grand Island, NY, USA, cat. 16000-044), 10 ng/ml EGF, 10 μg/ml FSH, 1 μg/ml ostradiol-17β, 0.6 mM cysteine, and 0.2 mM sodium pyruvate (Gibco BRL, Life Technologies, Grand Island, NY, USA, cat. 11360-070). Incubator conditions were set to 38.5°C and 5% CO_2_ for 22–24 h.

### *In vitro* Fertilization and Embryo Culture

*In vitro*-matured (MII) COCs were co-cultured with frozen–thawed bovine sperm of already proven fertility (Idrees et al., 2019a). The sperm straw was thawed at 37.0°C for 1 min and diluted in D-PBS. Subsequently, the sperm suspension was centrifuged at 750 × *g* for 5 min at room temperature, and the pellet was re-suspended in 500 μl of heparin (20 μg/ml) diluted in *in vitro* fertilization (IVF) medium [Tyrode's lactate solution supplemented with 6 mg/ml bovine serum albumin (BSA), 22 μg/ml sodium pyruvate, 100 IU/ml penicillin, and 0.1 mg/ml streptomycin]. The heparin-diluted sperm suspension was incubated at 38.5°C and 5% CO_2_ for 15 min to facilitate capacitation. Subsequently, the sperm suspension was diluted in IVF medium to a final density of 1.0–2.0 × 10^6^ sperms/ml and co-cultured with mature oocytes for 20 h. On the next day, the cumulus cells were removed from the presumed zygotes by vortexing for 3 min and cultured in SOF-BE1 medium supplemented with 5 μg/ml insulin, 5 μg/ml transferrin, 5 ng/ml sodium selenite (cat. 11074547001), 4 mg/ml fatty-acid-free BSA, and 100 ng/ml EGF. The presumed zygotes were cultured for 8 days, with one-time media replacement after 3 days.

### RNA Extraction and Reverse Transcription Quantitative PCR

To generate cDNA, mRNA was extracted from samples using the Dynabeads mRNA Direct Kit (Dynal AS, Oslo, Norway) according to the manufacturer's protocol and as previously described (Idrees et al., 2019b). The concentration of the purified mRNA was determined spectroscopically (NANO DROP 2000c Thermo Fisher Scientific) at 260 nm. Superscript III reverse transcriptase (iScript® cDNA Synthesis Kit from Bio-Rad Laboratories Hercules, CA, USA) was used to reverse-transcribe the first-strand cDNA. The PCR primers were designed using the Primer3 (v. 0.4.0) software of the National Center for Biotechnology Information nucleotide database. A CFX98 instrument (Bio-Rad Laboratories) was used to quantify the transcription levels. The reaction was carried out in a final volume of 10 μl, comprising 3 μl diluted cDNA, 1X iQ SYBR Green Super mix (iQ SYBR Green Super Mix Kit, Bio-Rad Laboratories, cat. 170-8882 Laboratories Hercules, CA, USA), and 0.2 mM each of forward and reverse primers. The samples were processed in triplicate according to the manufacturer's guidelines. The relative gene expression was calculated using the threshold ^ΔΔ^C(t) method. The *GAPDH* gene was used as an endogenous control and for the normalization of the expressed data. The primers and the PCR conditions for each gene are given in Supplementary Table 4.

### H_2_DCFDA Staining for Reactive Oxygen Species

To measure the reactive oxygen level (ROS) level, 2,7-di-chloro-di-hydro-fluorescein di-acetate (H2DCFDA, cat. #D6883) staining was performed according to a previously described protocol (Idrees et al., 2019a). In brief, the MII stage live oocytes were incubated in PBS containing 10 nM H2DCFDA for 30 min at 38.5°C and 5% CO_2_. After that, the samples were washed thrice with PBS and examined under an epifluorescence microscope (Olympus IX71) under 490-nm excitation and 525-nm emission filters.

### Cells and Cell Culture

#### Primary Bovine Cumulus Cell Culture

Bovine cumulus cells obtained from 3–6-mm COCs were cultured in previously described media and conditions (Baufeld and Vanselow, 2013). In brief, cumulus cells were collected immediately after COC isolation and denuded by recurrent pipetting through a narrow pipette. The collected cumulus cells were cultured on collagen-coated 24-well-plates with 1.25 × 10^5^ viable cells per well. The serum free α-MEM medium was supplemented with HEPES (20 mM), BSA (0.1%), sodium bicarbonate (0.084%), sodium selenite (4 ng/ml), transferrin (5 μg/ml), insulin (10 ng/ml), androstenedione (2 μM), L-glutamine (2 mM), non-essential amino acids (1 mM), penicillin (100 IU), and streptomycin (0.1 mg/ml). FSH (20 ng/ml) and EGF (25 ng/ml) were also added to the culture medium to stimulate the cells.

### COV434 Cell Culture, Treatment, and Transfection

The COV434 human granulosa cell line was kindly gifted by Professor Jeehyeon Bae (Chung-Ang University, Seoul, Republic of Korea). Cells were cultured in Dulbecco's modified Eagle's medium containing 1% penicillin–streptomycin and 10% FBS as previously described (Jin et al., 2016). The incubator conditions were set to 37.0°C and 5% CO_2_ air. Cell treatment was performed using the MTT assay in accordance with the manufacturer's protocol (Sigma-Aldrich). In 96-well-plates, COV434 cells were cultured at a concentration of 0.8 × 10^4^ cells per well, with 200 μl of media already contained per well. After growing the cells for 24 h, the medium was replaced with a fresh medium containing no or varying concentrations of PHPS1 and incubated for 24 h. Subsequently, the cells were incubated with MTT solution for 3 h. Thereafter, DMSO (100 μl) was added, and the plate was agitated for 20 min. A microplate scanning reader was used to measure absorbance at 550 to 570 nm (L1, value for viable cells) and 620 to 650 nm (L2, value for debris). The corrected absorbance (*A* = L1–L2) was used to calculate the number of viable/dead cells in each well for each group.

Lipofectamine™ RNAiMAX Transfection Reagent (Thermo Fisher Scientific, Waltham, MA USA cat #13778030) was used to transiently transfect the COV434 cell line. Briefly, lipofectamine was diluted in the Opti-MEM medium (Thermo Fisher Scientific, Inc.), and the transfection complexes were prepared by diluting the siRNAs (Santa Cruz products sc-37007 and sc-36488, respectively, Santa Cruz Biotechnology, St. Louis, MO, USA) in the Opti-MEM medium. The diluted siRNA was added to the diluted lipofectamine reagent and incubated for 20 min at room temperature. The transfection complexes were added to each well and incubated for 24 h, after which the medium was replaced with a fresh one. At 48 h after transfection, the cells were fixed (4% paraformaldehyde) or lysed for the examination of specific protein expression *via* immunoblotting or immunofluorescence.

### Co-immunoprecipitation and Antibodies

To analyze the SHP2/ERα complex, co-immunoprecipitation was performed according to the manufacturer's protocol (Pierce Co-IP cat. 26149). The SHP2 antibody was used to pull down the protein complex. The SHP2/ERα complex levels in various samples were analyzed *via* western blotting. The following primary antibodies were used in this study: β-actin (Santa Cruz cat. sc-47778), SHP2 (Santa Cruz Biotechnology, USA cat. sc-271106), ERα (Abcam, Cambridge, Cambs, UK cat. ab3575), AKT (Cell Signaling technology cat. 9272), p-AKT (Cell Signaling, cat. 9271), p-ERK1/2 (Cell Signaling, cat. CST 9101S), ERK1/2 (Cell Signaling, cat. CST 9102S), FOXL2 (LifeSpan BioScience, Seattle, WA, USA cat. LS-B12865), SF1 (Abcam cat. ab168380), Wnt4 (Santa Cruz cat. sc-376279), octamer-binding transcription factor 4 (OCT4; Santa Cruz cat. Sc-8629), Caspase-3 (Santa Cruz cat. Sc-1225), p-Nf-Kb (Santa Cruz cat. Sc-271908), and p-mTOR (Abcam cat. ab84400). The secondary antibodies used in this study were mouse horseradish peroxidase (HRP; Amersham ECL cat. NA931), rabbit HRP (Amersham ECL cat. NA934), mouse-FITC (Santa Cruz cat. sc-516140), and rabbit-TRITC (Thermo Fisher Scientific, Waltham, MA, USA, cat. A16101).

### Histological Analysis

Bovine ovaries collected from the local abattoir were washed with saline and stored at 4°C in 20% sucrose diluted in 1× PBS solution for 72 h. Thereafter, the ovaries were washed with saline and stored in 4% paraformaldehyde at 4°C for 72 h. The ovaries were placed in an optimal cutting temperature compound (Sakura Finetek Inc., Torrance, CA, USA) and stored at −80°C for a minimum of 24 h to make blocks, and 12-μm-thick sections were taken on probe-on plus charged slides (Fisher, Rock-ford, IL, USA) using a CM 3050C cryostat (Leica, Germany). The slides were stored at −80°C until further processing.

### Immunofluorescence

For immunofluorescence, staining was performed according to a previously defined protocol (Idrees et al., 2019b). In brief, 4% (v/v) paraformaldehyde in 1 M PBS was used to fix the samples, which were preserved at 4°C for a minimum of 30 min to a prolonged period. On the staining day, the samples were washed twice in 0.3% polyvinyl alcohol in 1× PBS (PBS-PVA) and permeabilized with the proteinase K solution (0.1%) for 5 min. After washing twice with PBS-PVA for 5 min, the samples were incubated for 90 min in 5% blocking solution (BSA-PBS-PVA). Primary antibodies were applied to the samples and kept at 4°C overnight. On the next day, after washing twice with PVA-PBS for 10 min, the samples were incubated with secondary antibodies (FITC and TRITC) at room temperature for 90 min. For nuclear staining, 10 μg/ml 4,6-diamidino-2-phenylindole was applied for 5 min. Thereafter, the samples were washed thrice with PVA-PBS for 5 min, mounted with a fluorescent mounting medium, and covered with a slide cover. A confocal laser scanning microscope (Fluoview FV 1000, Olympus, Japan) was used to capture images. ImageJ analysis software (National Institutes of Health, Bethesda, MD, USA; https://imagej.nih.gov/ij) was used to measure the relative integrated density of the signals.

### Immunoblotting

The samples [MII oocytes (100 per extract) or trypsinized cells] were washed with D-PBS and dissolved in PRO-PREP™ (iNtRON Biotechnology, Burlington, NJ, USA, cat. 17081). Subsequently, the samples were sonicated, and the cell lysate was centrifuged at 10,000 × *g* for 25 min at 4°C. The supernatant was collected and quantified using the Bradford assay (cat. 5000002 Laboratories Hercules, CA, USA), while the settled debris was discarded (Idrees et al., 2019a). Equal amounts of protein (10–20 μg) were fractionated by SDS-PAGE (10 and 12%) and then transferred to a polyvinylidene fluoride (PVDF) membrane (cat. GE 10600023; Sigma-Aldrich). The PVDF membrane was blocked with skimmed milk for 1 h and incubated overnight with a primary antibody at 4°C in a two-dimensional shaker. Thereafter, it was incubated with HRP-conjugated secondary antibody at room temperature for 90 min. An enhanced chemiluminescence detection reagent (Pierce TM ECL Western Blotting Substrate, Thermo Fisher Scientific, Waltham, MA, USA) was used to detect bound antibodies. Protein ladders (Abcam, USA, cat. ab116029) were used to determine the molecular weights of the proteins. ImageJ software (National Institutes of Health, Bethesda, MD, USA; https://imagej.nih.gov/ij) was used to detect the optical densities of the bands on X-ray films (iNtRON, Biotechnology Inc., Burlington, NJ, USA).

### Molecular Docking of ERα and SHP2

The 3D structural information on the binary complex of human ERα and SHP2 has not been reported yet; therefore, we performed a molecular docking study to gain an insight into the molecular-level interaction between ERα and SHP2. The ERα complex structure was not available in Protein Data Bank (PDB), but a recent study modeled a full-length structure of the ERα comprising the ligand-binding domain and DNA-binding domain, which is available in the Small-Angle Scattering Biological Data Bank (Valentini et al., 2015; Huang et al., 2018). For SHP2, an open conformation structure (PDB ID: 6CRF) was selected from the literature (LaRochelle et al., 2018) and downloaded from PDB (https://www.rcsb.org/). The structures of both proteins were prepared using a clean protein protocol and were further minimized using the Discovery Studio v2018 software (http://3dsbiovia.com/products/). Water molecules were removed, and hydrogen atoms were added. For molecular docking, we utilized HDOCK, the web server for protein–protein and protein–DNA/RNA docking (Yan et al., 2020). During molecular docking, ERα was used as a receptor and SHP2 as a ligand. Owing to the lack of structural information for the ERα and SHP2 complex, the docking simulation was performed with default parameters, without defining the binding site according to a previously established method (Selent et al., 2013). The HDOCK server uses a fast Fourier transform-based approach to sample all possible binding modes globally (Katchalski-Katzir et al., 1992). Additionally, all binding modes collected were reassessed with iterative-knowledge-based scoring functions (Yan et al., 2017).

### Molecular Dynamic Simulation for the ERα and SHP2 Binary Complex

The prediction of the correct 3D binding mode for protein–protein interaction is extremely difficult, as protein-protein molecular docking studies have always been challenging. Therefore, to refine the docked ERα-SHP2 complex, we subjected the best docked structure to molecular dynamics (MD) simulations using GROMACS v2018.2 (Pronk et al., 2013). The topology parameters of the protein were prepared by the AMBER99SB-ILDN force field (Lindorff-Larsen et al., 2010). The binary complex was placed into a dodecahedron box, and the TIP3P water model was used for solvation. The system was neutralized by 18 Na^+^ ions and subjected to an energy-minimization step with the steepest descent algorithm to avoid steric clashes and bad contacts. Subsequently, the energy-minimized system was subjected to equilibration in two phases. In the first phase, temperature balancing was performed under an NVT ensemble for 500 ps at 300 K while using a V-rescale thermostat (Bussi et al., 2007). In the second phase, pressure equilibration was achieved under the NPT ensemble for 500 ps at 1.0 bar using the Parrinello–Rahman barostat (Parrinello and Rahman, 1981). Each system was then subjected to 50 ns of production run. The particle mesh Ewald method was used to estimate the electrostatics of long-range interactions (Darden et al., 1993). During simulation, the bond lengths were restrained using the Linear Constrain Solver algorithm (Hess et al., 1997). The MD simulation results were analyzed using the visual molecular dynamics (Humphrey et al., 1996) and DS software programs. Additionally, the binding free energy calculations were performed with the molecular mechanics-generalized Born surface area (MM-GBSA) algorithm using an online webserver, Hawk Dock (Weng et al., 2019).

### Statistical Analysis

At least three separate and independent experiments were performed to derive data, which were expressed as mean ± SEM. The western blotting bands and immunofluorescence images were analyzed using Graph Pad Prism 6 (Graph Pad Software, USA) and Image J software programs. *P-*values were calculated on the basis of one-way analysis of variance (ANOVA), followed by Student's *t*-test (significance: ^*^p < 0.05, ^**^p < 0.01, ^***^p < 0.001, and ^****^p < 0.0001).

## Results

### SHP2 Expression in Ovarian Tissue and Dynamic Changes With Growth Factor Treatment in Cultured Bovine Granulosa Cells

**Figure 1:**
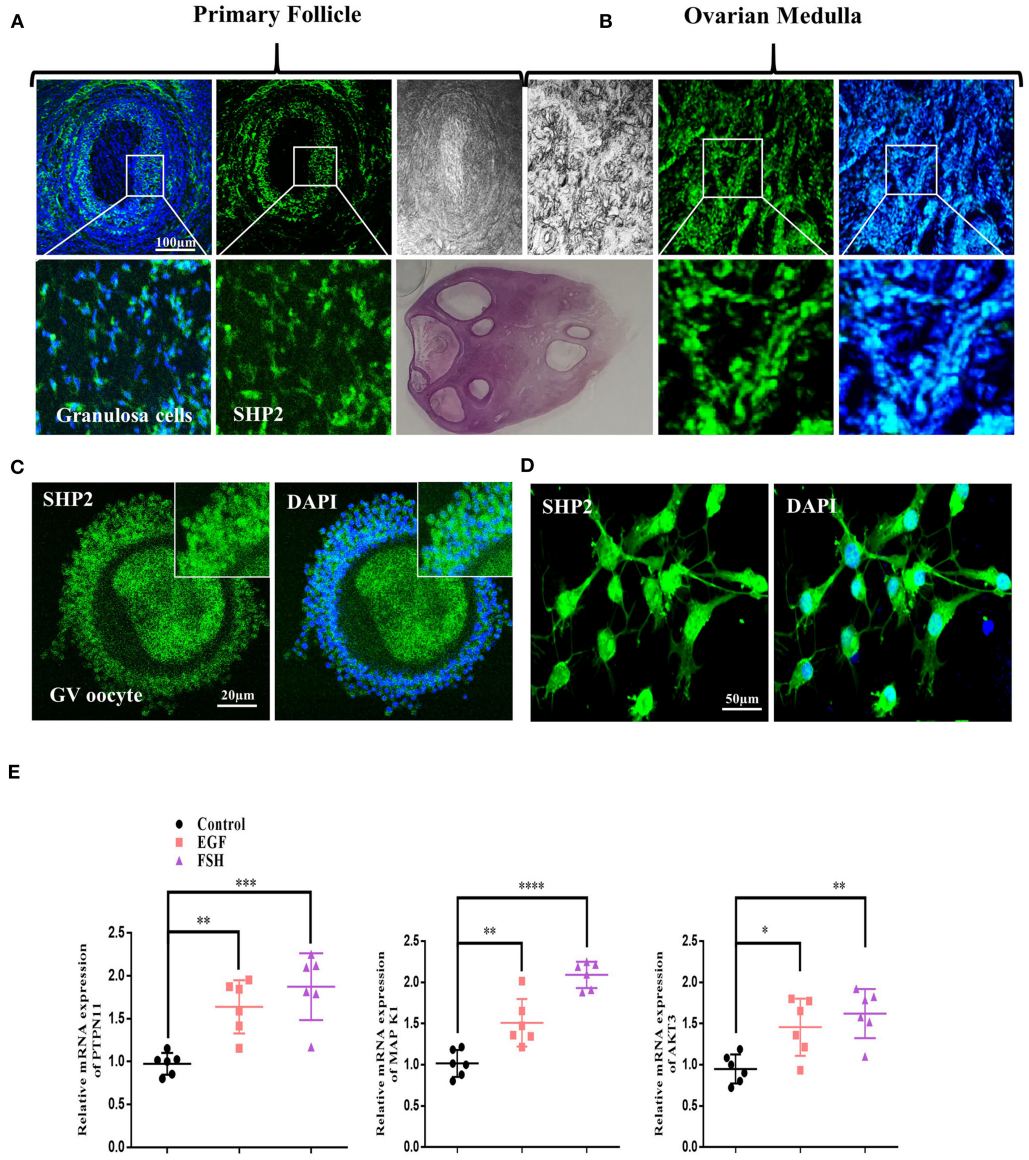
Src-homology-2-containing phosphotyrosine phosphatase (SHP2) expression in bovine ovary and upregulation with follicle-stimulating hormone (FSH) or epidermal growth factor (EGF) treatment. **(A)** Representative images of SHP2 protein expression (green, fluorescein isothiocyanate) in bovine pre-ovulatory oocyte granulosa cells. **(B)** SHP2 protein expression in bovine ovarian medulla. The lower images are zoom-ins of the upper images. **(C)** SHP2 expression in the cumulus cells of bovine oocyte (germinal vesicle, GV). **(D)** SHP2 expression in cultured cumulus cells obtained from GV-stage oocytes. **(E)** Cultured cumulus cells treated with FSH or EGF and PTPN11 (*SHP2*), mitogen-activated protein kinase 1 (*MAPK1*), and protein kinase B (*AKT3*) genes analyzed *via* RT-qPCR (the experiment was repeated thrice, and data are shown as mean ± SEM. NS, not significant; *P < 0.05; **P < 0.01; ***P < 0.001; ****P < 0.0001.

To investigate the protein localization pattern of SHP2 in the somatic compartment of a follicle, we first performed the immunofluorescence staining of bovine ovarian sections with an SHP2-specific antibody (Figures 1A,B; Eppig and Handel, 2012). The results showed the protein expression of SHP2 in the granulosa cells of pre-ovulatory follicles and in the ovarian medulla. The localization pattern analysis revealed that SHP2 was evenly distributed between the nucleus and the cytosol. To analyze SHP2 localization in cumulus cells, the ovarian immature COCs were fixed and immunofluorescent-stained with the SHP2 antibody (*n* = 20 COCs per group) (Figure 1C). The results showed that SHP2 had a localization pattern similar to that identified in granulosa cells. To further confirm the nuclear localization of SHP2, pre-antral-follicle-derived bovine cumulus cells were cultured *in vitro*, and SHP2 was evaluated (Figure 1D; Georges et al., 2014). Although SHP2 is a main regulating component of growth factor receptor intracellular signaling, it showed unconventional nuclear localization in granulosa and cumulus cells. To examine the function of SHP2, cultured cumulus cells were treated with EGF and FSH, and samples were prepared for RT-qPCR (Figure 1E). The mRNA expression of protein tyrosine phosphatase N11 (*PTPN11* encoding protein SHP2) and *AKT3* genes was found to be statistically significant with EGF or FSH stimulation as compared to the control, while *MAP K1* was found to be very significant with FSH treatment (*n* = 20 per group; ^*^p < 0.05, ^**^p < 0.01, ^****^p < 0.0001). These findings indicate that SHP2 shows nuclear and cytoplasmic localization in bovine granulosa and cumulus cells. EGF or FSH treatment can upregulate *PTPN11* (SHP2) mRNA expression in cultured cumulus cells (Dance et al., 2008), but the function of the nucleus-localized SHP2 still needs exploration.

### SHP2 Forms a Complex With ERα in GV Oocyte Cumulus Cells and Is Involved in ERα Transcriptional Activity

**Figure 2:**
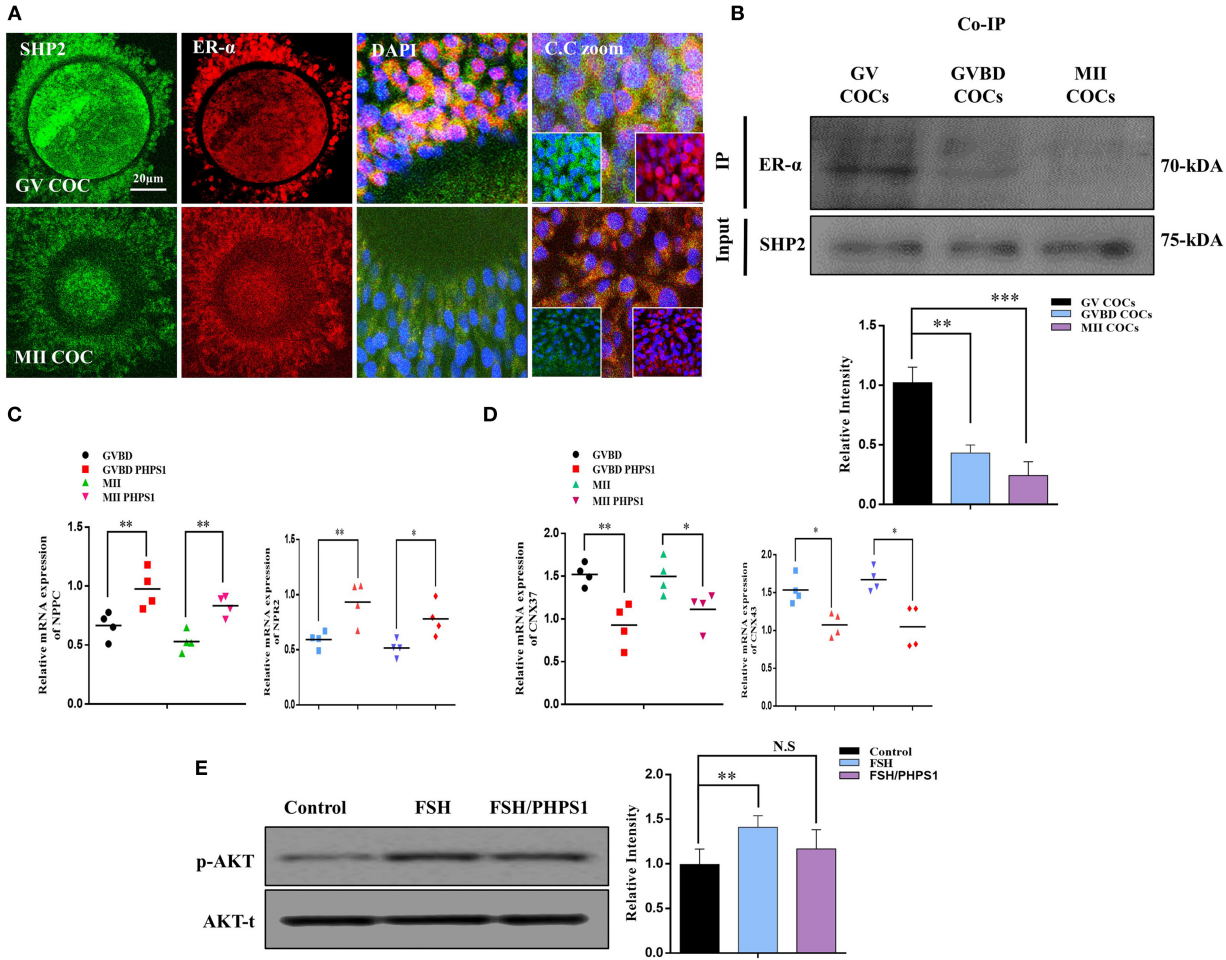
Src-homology-2-containing phosphotyrosine phosphatase (SHP2)/estrogen receptor alpha (ERα) complex and ERα-dependent gene expression in cumulus cells. **(A)** SHP2 and ERα proteins analyzed in germinal vesicle (GV)- and MII-stage cumulus–oocyte complexes (COCs) *via* immunofluorescence. Both proteins showed nuclear localization and exited the nucleus with the progression of meiotic maturation. Data are expressed as means ± SEM. GV and MII COCs were immune-labeled with SHP2 (green) and ERα (red) and counterstained with 10 μg/ml 4′,6′-diamidino-2-phenylindole to visualize DNA (mean ± SEM). **(B)** Co-immunoprecipitation of SHP2 and ERα showed that both proteins form a complex at the GV COC stage, and the complex is significantly reduced with oocyte maturation. **(C)** Natriuretic peptide C (*NPPC*) and natriuretic peptide receptor 2 (*NPR2*) genes analyzed *via* RT-qPCR in GV, germinal vesicle breakdown (GVBD), and MII-stage COCs and compared with phenylhydrazonopyrazolone sulfonate 1 (PHPS1) treatment of GVBD- and MII-stage oocytes. **(D)** *CX37* and *CX43* genes analyzed *via* RT-qPCR in GVBD- and MII-stage COCs and compared with the PHPS1 treatment of GVBD- and MII-stage oocytes. **(E)** Cultured bovine granulosa cells were stimulated with follicle-stimulating hormone (FSH) and FSH plus PHPS1, and the p-AKT protein was analyzed along with total AKT. The experiments were repeated thrice, and data are shown as mean ± SEM. NS, not significant; *P < 0.05; **P < 0.01; ***P < 0.001; ****P < 0.0001.

ERα plays a key role in oocyte meiotic resumption and maturation, and several studies have identified the role of nuclear SHP2 in the transcriptional activity of ERα (Li et al., 2014; Ran et al., 2017). To analyze the interaction of SHP2 and ERα in the cumulus cells of GV- and MII-stage oocytes, we immunostained COCs with specific SHP2 and ERα antibodies (Figure 2A). At the GV stage, SHP2 and ERα were localized in the nucleus of cumulus cells, but at the MII stage, both proteins showed complete cytoplasmic localization. Few studies have identified that SHP2 forms a complex with ERα in the MCF7 and Ishikawa cell lines for activating signaling pathways and ERα transcription (Li et al., 2014; Ran et al., 2017). To understand the SHP2 and ERα behavior in cumulus and granulosa cells, samples were collected from ovary-retrieved immature COCs, 16-h cultured germinal vesicle breakdown (GVBD) COCs, and 22-h cultured mature (MII) COCs for the Co-IP of SHP2 and ERα proteins (Figure 2B). These data imply that SHP2 exists in complex with ERα in immature COCs, but the amount of complex becomes significantly reduced with the progression of meiotic maturation. It was previously identified that ERα binds with the promoter regions of *NPPC* and *NPR2* in granulosa and cumulus cells and sustains oocyte meiotic arrest (Liu et al., 2017). Therefore, we applied PHPS1, a site-specific inhibitor of SHP2, to examine ERα transcriptional activity. Contrary to our expectations, the PHPS1-treated COCs showed a higher expression of *NPPC/NPR2* as compared to the control COCs, in which the expression of both genes was significantly reduced with meiotic progression (*n* = 20 per group; ^*^P < 0.05, ^**^P < 0.01; Figure 2C). To further confirm, we analyzed the expression of connexin-43 (CX43) and connexin-37 (CX37), as both genes are required for *NPPC/NPR2* signaling to mediate oocyte meiotic arrest (Richard and Baltz, 2014). The results indicated a significantly higher expression of both *CX37* and *CX43* in the control group than in the PHPS1-treated COCs (Figure 2D; *n* = 20 per group; ^*^P < 0.05, ^**^P < 0.01). FSH activates MAP kinases and AKT signaling in the cumulus cells of oocytes, which inhibit *NPPC/NPR2* (Tsuji et al., 2012; Wang et al., 2013), while SHP2 inhibition reduced FSH signaling in granulosa cells (Donaubauer et al., 2016). We found that FSH stimulation of cultured cumulus cells enhanced the expression of p-AKT protein, whereas the combined application of FSH and PHPS1 significantly reduced its expression (^*^P < 0.05, ^**^P < 0.01; Figure 2E). All the above-mentioned results revealed that SHP2 exists in complex with ERα, and this complex shows a significant reduction with the progression of oocyte meiotic maturation. Unexpectedly, SHP2 inhibition had no significant effect on ERα transcriptional activity during COC meiotic maturation, but it can significantly reduce FSH intracellular signaling in cultured cumulus cells.

### Cumulus Cell Cytoplasmic SHP2 Inhibition Deteriorates Oocyte Maturation and Embryo Development

**Figure 3:**
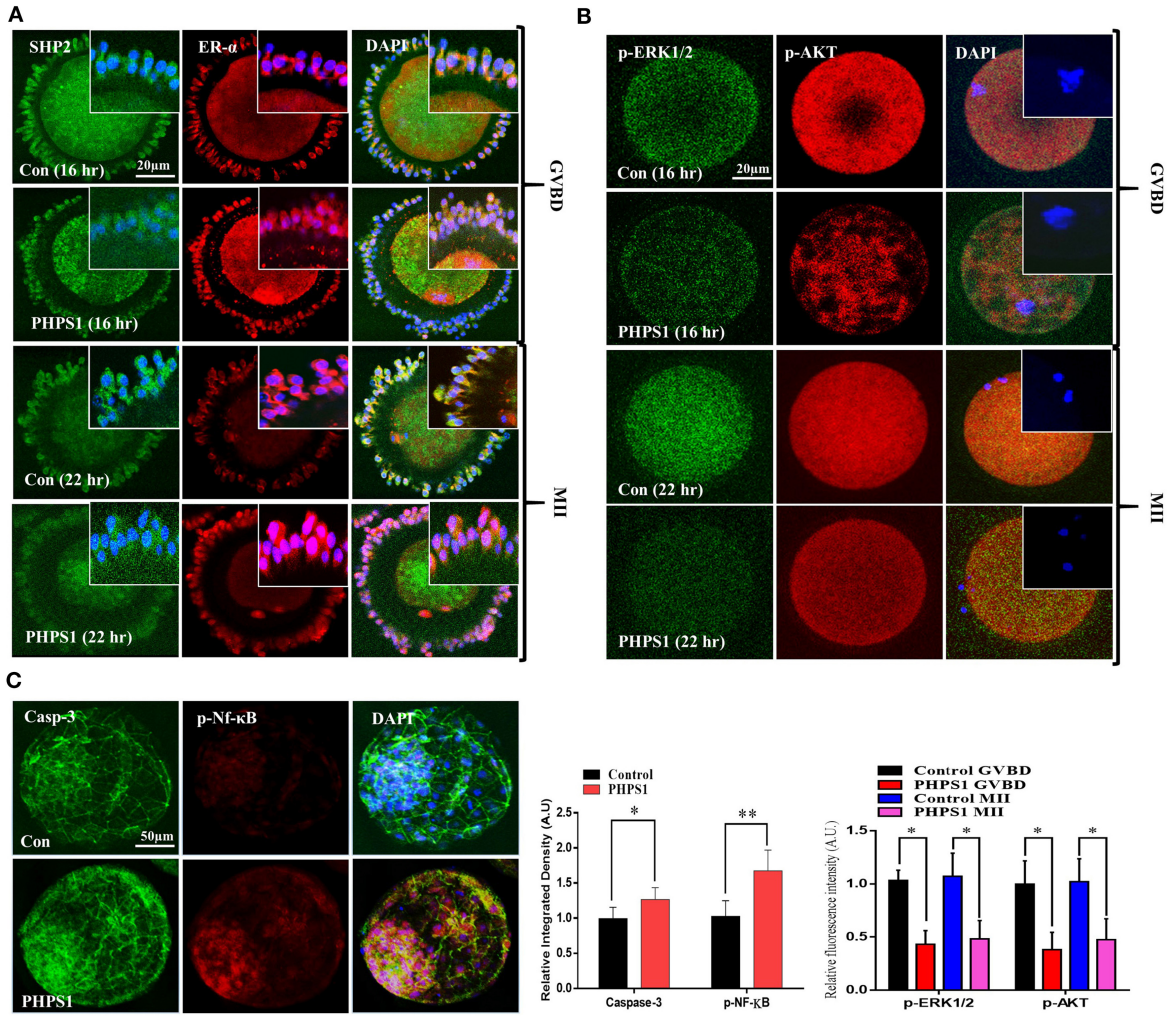
Cumulus-cell cytoplasmic Src-homology-2-containing phosphotyrosine phosphatase (SHP2) inhibition deteriorates oocyte maturation and embryo development. **(A)** SHP2/ERα (GFP-labeled SHP2 and red fluorescent protein-labeled ERα) proteins analyzed *via* immunofluorescence in cumulus cells during germinal vesicle breakdown (GVBD) and MII stages of control cumulus–oocyte complexes (COCs) and phenylhydrazonopyrazolone sulfonate 1 (PHPS1)-treated COCs. **(B)** Representative images of p-ERK1/2 and p-AKT in GVBD- and MII-stage oocytes in the control and PHPS1-exposed groups. 4′,6′-Diamidino-2-phenylindole was used to stain the nucleus. **(C)** Immunofluorescence images of Caspase-3 and phosphorylated NF-Kb in control and PHPS1-treated bovine day 8 blastocysts. Qualitative analysis showed that PHPS1 treatment enhances p-NF-kB nuclear localization and enhances Caspase-3 expression. All experiments were repeated thrice, and data are shown as mean ± SEM. NS, not significant; *P < 0.05; **P < 0.01; ***P < 0.001.

SHP2 cytoplasmic localization is essential for FSH-induced activation of MAP kinases and AKT signaling, and PHPS1 inhibits active cytoplasmic SHP2 (Donaubauer et al., 2016; Idrees et al., 2019b). To analyze the effects of FSH signaling blockage *via* SHP2 inhibition on oocyte meiotic maturation, we treated immature COCs with PHPS1 and collected samples for immunofluorescence with the progression of meiotic stages [oocyte maturation percentage control *n* = 172 (84%) *vs*. PHPS1 *n* = 185 (58%), *n* = 50 per group, with four independent biological replicates] (Figure 3A). The PHPS1-treated COCs showed reduced cytoplasmic localization of SHP2 and ERα compared to the control, where both proteins were localized in the cytoplasm (*n* = 30 per group; ^*^P < 0.01). Next, we tested p-ERK1/2 and p-AKT signals in the MII oocytes (Figure 3B), as both proteins are essential for oocyte meiotic maturation and are downstream of SHP2 (Hatch and Capco, 2001; Andrade et al., 2017). We found a significant reduction in the p-ERK1/2 and p-AKT proteins in GVBD and MII oocytes treated with PHPS1 compared to control oocytes. Furthermore, the MII oocyte also showed a significant enhancement in ROS (Supplementary Figure 1A). Given the effects of SHP2 inhibition on oocyte quality, we speculated whether the loss of SHP2 would impair the development of subsequent embryos. To do this, we carried out IVF using control and SHP2-inhibited oocytes, and then zygotes were cultured in IVC media for 8 days to check the development. PHPS1 application reduced the embryo development by almost 50% [control *n* = 143 (39%) *vs*. PHPS1 *n* = 108 (21%)]. The SHP2-inhibited oocytes that developed to the blastocyst stage showed significantly high apoptosis as identified *via* nucleus-localized NF-kB and enhanced caspase-3 expression (Figure 3C). Furthermore, the developed blastocysts showed the reduced expression of OCT4 (Supplementary Figure 1B; Simmet et al., 2018). Moreover, SHP2 downstream proteins p-AKT and p-ERK1/2 showed a significant reduction with PHPS1 treatment as compared to the control littermates (Supplementary Figure 1C). These findings altogether suggest that SHP2-inhibited oocytes, in many cases, are unable to develop properly.

### FSH Reduces SHP2/ERα Complex in Human Granulosa Cells (COV434)

**Figure 4:**
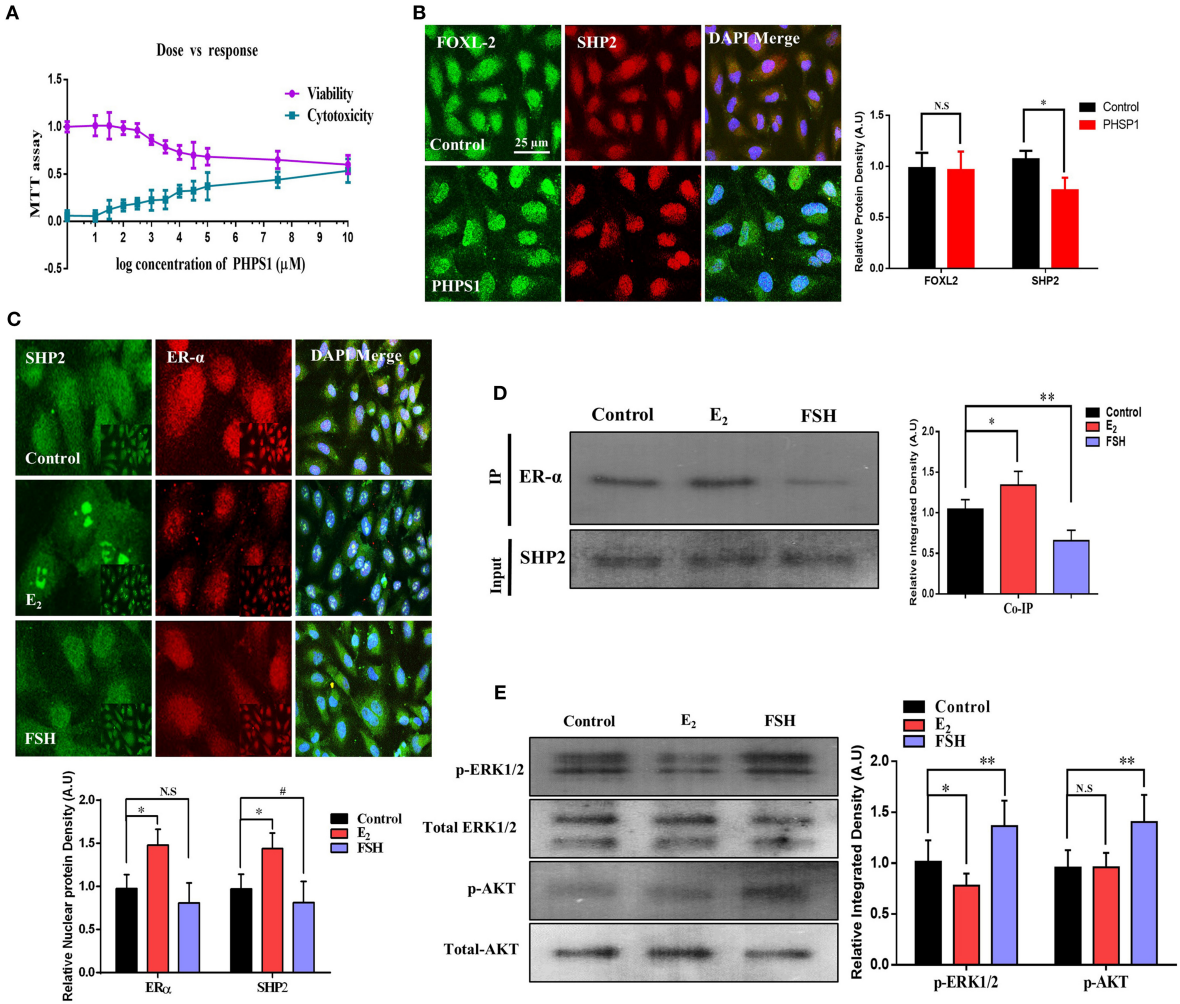
Follicle-stimulating hormone (FSH) reduces the amount of SHP2/ERα complex in human granulosa cells (COV434). **(A)** To obtain the effective concentration of phenylhydrazonopyrazolone sulfonate 1 (PHPS1), an MTT assay was performed in COV434 cells, and 3 μM of PHPS1 was identified as the least effective concentration. **(B)** Immunofluorescence experiment of FoxL2 (green) and SHP2 (red) identified that SHP2 shows nuclear localization in COV434 cells, and PHPS1 had no effect on SHP2 nuclear localization. **(C)** FSH- and E_2_-stimulated COV434 cells showed the differential localization of SHP2 and ERα after 24 h. Estradiol (E_2_) significantly enhanced the nuclear localization of SHP2, while FSH significantly reduced its nuclear localization, compared to that in the control group. **(D)** Co-immunoprecipitation identified that SHP2 and ERα form a complex in COV434 cells, and E_2_ or FSH treatments had a significant effect on this complex. **(E)** FSH augmented extracellular signal-regulated kinase (ERK) and AKT signaling in the COV434 cell line compared to that in the control and E_2_-treated groups, wherein signaling was significantly reduced. The experiments were repeated thrice, and data are shown as mean ± SEM. NS, not significant; *P < 0.05; **P < 0.01; ***P < 0.001.

To uncover the SHP2/ERα complex expression, localization, and involvement in human oocyte meiosis, we cultured an immortalized human granulosa cell line COV434 that exhibits the biological characteristics of normal human granulosa cells (Zhang et al., 2000). Granulosa cells were treated with PHPS1 at various concentrations, and 3.5 μM was selected as the least effective concentration *via* an MTT assay (Figure 4A). Thereafter, SHP2 and fork head box L2 (FOXL2, a surrogate marker of granulosa cells) antibodies were applied, and the results showed that SHP2 had a localization pattern similar to that previously detected in cultured bovine cumulus cells (Figure 4B; Georges et al., 2014). PHPS1 application to human granulosa cells had no noticeable effect on nucleus-localized SHP2. To analyze the effects of estradiol and FSH on SHP2 and ERα localization in granulosa cells, we applied E_2_ (10.0 μM) and FSH and checked both proteins *via* immunofluorescence (Figure 4C). The results indicated that the application of E_2_ highly localized SHP2 in the nucleus and that of FSH translocated it to the cytoplasm. To detect the SHP2/ERα complex in granulosa cells and determine the effects of E_2_ and FSH on this complex, we performed a co-immunoprecipitation assay (Figure 4D). The SHP2/ERα complex was detected in the control group and was significantly enhanced with E_2_, but the FSH treatment markedly reduced this complex in granulosa cells. The reduction in the SHP2/ERα complex caused by the FSH treatment might be due to FSH intracellular signaling in granulosa cells (Li et al., 2014). Therefore, the p-ERK1/2 and p-AKT proteins were analyzed *via* western blotting, and the results showed a high expression of these proteins in the presence of FSH as compared to the control and E_2_-treated groups (Figure 4E). The above-mentioned results indicated that the SHP2 and ERα complex is present in human granulosa cells, and the FSH treatment can reduce the amount of this complex. Furthermore, E_2_ acts in an opposite manner by enhancing the SHP2/ERα complex and reducing FSH-induced intracellular signaling in the COV434 cell line.

### Molecular Modeling Studies for Predicting the Binding Mode Between ERα and SHP2

**Figure 5:**
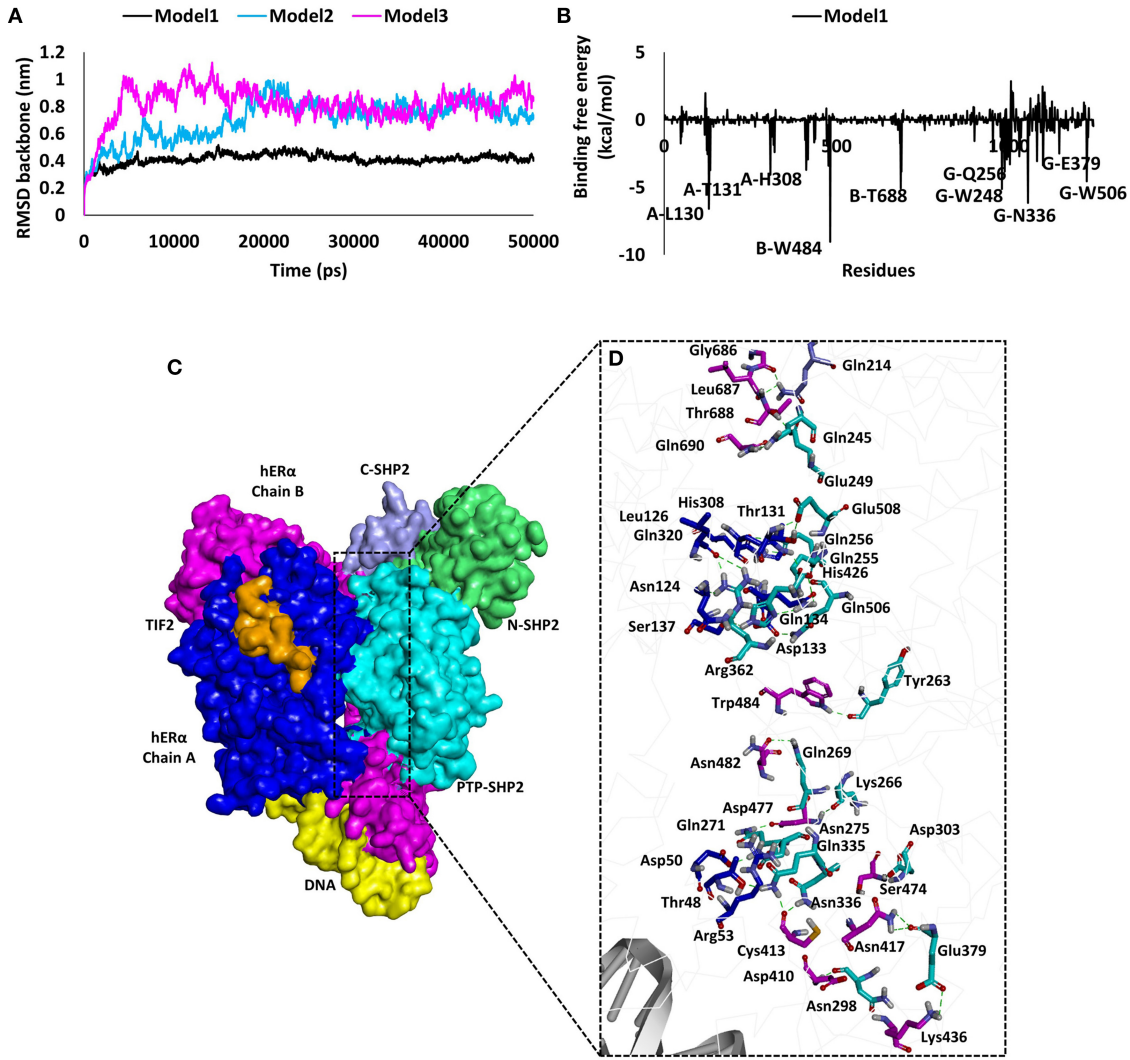
Results of molecular modeling studies for predicting the binding mode between human ERα and SHP2. **(A)** The root mean square deviation values of the backbone atoms of ERα and SHP2 complexes during the molecular dynamics simulation times. **(B)** Decomposition of the molecular mechanics-generalized Born surface area energy for each amino acid of the binding surface from ERα and SHP2. The key interacting residues observed in ERα and SHP2 are highlighted. **(C)** Predicted binding mode of ERα and SHP2 complex in surface representation. ERα chain A is shown in blue, chain B in pink, TIF2 in orange, and DNA in yellow. The SHP2 protein domains are represented in purple (C-SHP2), green (N-SHP2), and cyan (PTP domain). **(D)** Enlarged view for the contact area between the two proteins. Interacting amino acids are shown in stick representation. The pink (chain B) and blue (chain A) sticks are from ERα, whereas the cyan (PTP domain) and purple (C-SHP2) sticks are from the SHP2 protein. The hydrogen bonds are shown as dashed green lines.

To support our experimental findings, protein–protein docking studies were performed to determine the probable binding mode between human ERα and SHP2 proteins. For this purpose, protein–protein/DNA docking was performed using an online webserver, HDOCK. The 50 models generated were downloaded, and the binding modes of all models were then assessed by cluster analysis in Discovery Studio. The cluster analysis revealed that three different binding modes could be possible for ERα and SHP2. The best model with the lowest docking energy score from each cluster was selected and subjected to 50 ns of MD simulation (Supplementary Table 1). The stability of the entire complex structure during the simulation times was analyzed by examining the root mean square deviation (RMSD) of the protein backbone atoms. It was observed that the RMSD values of model 1 was significantly lower than those of model 2 and model 3 (Figure 5A), implying that the structure of model 1 was more stable than the others during the simulation times. Moreover, the binding free energy calculations using the MM-GBSA algorithm was performed to further predict the binding affinity. It was found that model 1 showed the lowest binding free energy score (−115.2 kcal/mol) among the three models (model 2, −72.47 kcal/mol; model 3, −70.44 kcal/mol)] (Supplementary Table 2). The MM-GBSA calculations also provide the contribution of each amino acid in the binding. In model 1, the key residues involved in complex formation were Trp484, Leu130, Thr688, Thr131, and His308 of ERα and Asn336, Trp248, Gln506, Gln256, and Glu379 of SHP2 (Figure 5B). The per-residue contribution for ERα and SHP2 interaction was also calculated in model 2 and model 3 and is shown in Supplementary Figures 2, 3.

Furthermore, the binding mode for the ERα and SHP2 interaction was analyzed by calculating the average structure from the last 5 ns of the MD simulation trajectory for model 1, model 2, and model 3 (Figure S3). A detailed analysis revealed that, in model 1, the ERα and SHP2 complex was observed to form 24 hydrogen bonds, five electrostatic interactions, and five hydrophobic interactions (Figures 5C,D, Supplementary Table 3). The residues in chain A and chain B of ERα interact with the catalytic domain of SHP2 that has been reported to have a ligand-binding site ranging from 260 to 510 amino acids (Hellmuth et al., 2008). Model 1 displayed a higher number of molecular interactions in this region as compared to model 2 and model 3 (Supplementary Table 3). Finally, model 1 was selected as an ideal model for the ERα and SHP2 interaction because of its stable RMSD throughout the simulation, the lowest binding free energy, and the highest number of interactions. Similar approaches for final model selection have also been used in previous studies (Selent et al., 2013; Ge et al., 2018; Wu et al., 2020).

### SHP2 Knockdown Reduced ERα Transcription of *NPPC*/*NPR2* and Aromatase Activity of Human Granulosa Cells

**Figure 6:**
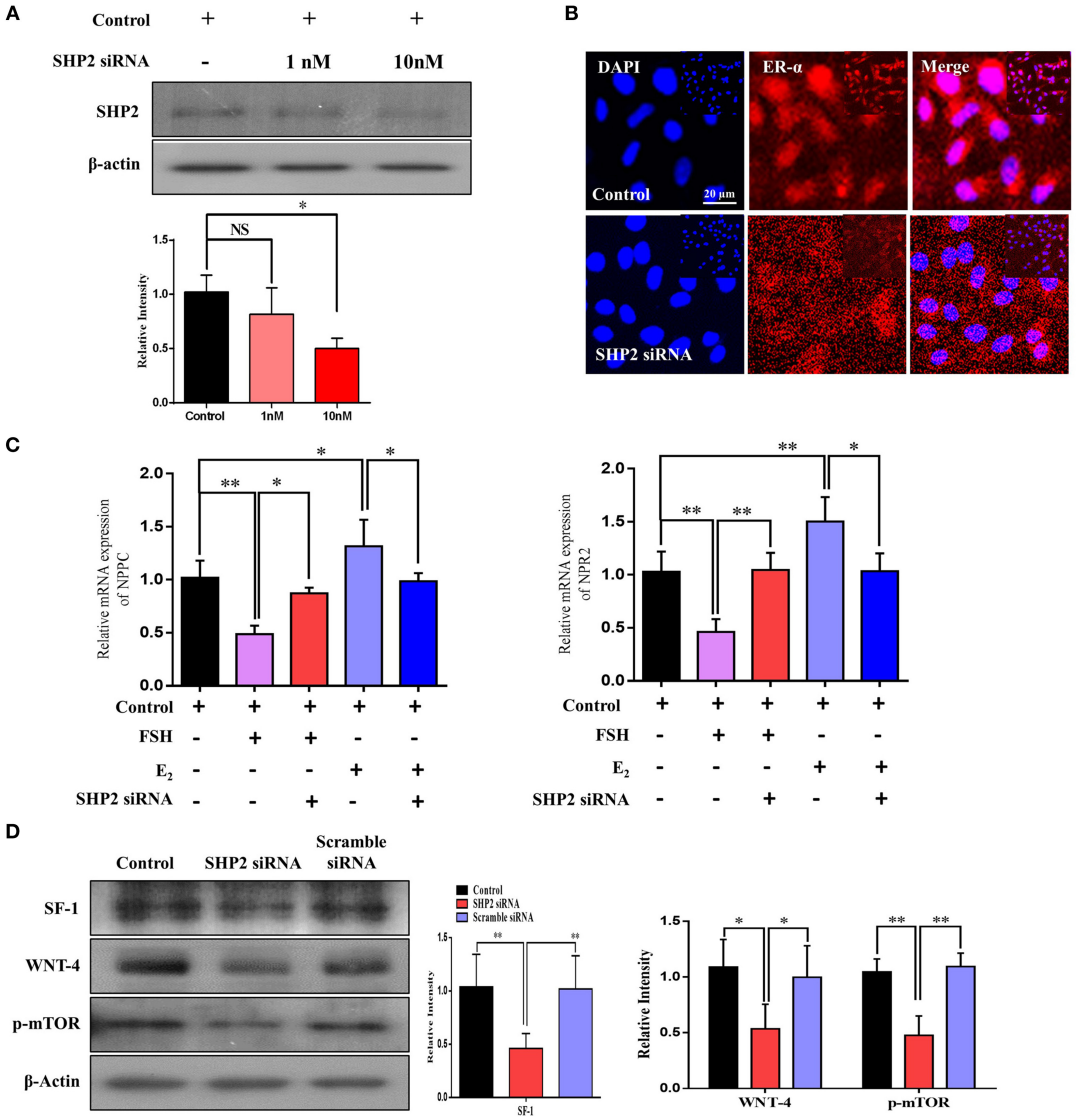
Src-homology-2-containing phosphotyrosine phosphatase (SHP2) knockdown reduced estrogen receptor alpha (ERα) transcription of the NPPC and NPR2 genes and aromatase activity of granulosa cells. **(A)** SHP2 siRNA was applied to COV434 cell line, and SHP2 protein expression was analyzed *via* western blotting. **(B)** To check the localization of ERα, an immunofluorescence experiment was performed using SHP2-knockdown COV434 cells. The results suggest that ERα nuclear localization was significantly reduced with SHP2 knockdown. **(C)** NPPC/NPR2 mRNA expressions were analyzed in follicle-stimulating hormone- and E_2_-stimulated SHP2-knockdown COV434 cell line. **(D)** To examine the effect of SHP2 knockdown on the functional activity of granulosa cells, the SF1, Wnt4, and p-mTOR proteins were analyzed *via* western blotting, and a significant reduction in all proteins was observed with SHP2 knockdown. β-Actin was used as the loading control for western blotting. Bands were quantified using ImageJ software, and the differences are represented by histograms. The experiments were repeated thrice, and data are shown as mean ± SEM. NS, not significant; *P < 0.05; **P < 0.01; ***P < 0.001.

To validate the direct link between the transcriptional activities of SHP2 and ERα in granulosa cells, we knocked down SHP2 *via* siRNA and verified it at the protein level (^*^P < 0.05; Figure 6A). SHP2-knockdown cells were immunofluorescent-stained with ERα antibody, and the results showed a significant reduction in ERα nuclear localization (Figure 6B). E_2_ and FSH have opposing regulatory effects on *NPPC*/*NPR2* genes in granulosa cells, and all of our above-mentioned results showed that SHP2 plays a role in the transcriptional activity of E_2_ and in the intracellular signaling of FSH. To directly analyze SHP2 interactions with E_2_ and FSH, the SHP2-knockdown granulosa cells were stimulated with E_2_ and FSH, and the mRNA expression of *NPPC*/*NPR2* was quantified *via* RT-qPCR (Figure 6C) (Liu et al., 2017). E_2_ application to granulosa cells had a significant effect on ERα transcriptional activity, while SHP2 siRNA abrogated the E_2_ stimulation of ERα and enhanced *NPPC*/*NPR2* mRNA expression (control *vs*. E_2_ vs. E_2_ + SHP2 siRNA; ^*^P < 0.05, ^**^P < 0.01). Furthermore, SHP2 knockdown interrupted FSH intracellular signaling-induced inhibition of *NPPC* and *NPR2* genes (control *vs*. FSH *vs*. FSH + SHP2 siRNA; ^*^P < 0.05, ^**^P < 0.01). Moreover, we examined the effect of SHP2 knockdown on the functional activities of granulosa cells, taking into account the potential interference with FSH signaling-mediated transcriptional factor activation. We found that SHP2 knockdown markedly reduced the steroidogenic factor-1 (SF1 encoded by the *NR5A1* gene) and β-catenin pathway protein wingless-type MMTV integration site family member 4 (Wnt4) proteins (Figure 6D) (control *vs*. siRNA *vs*. scramble siRNA; ^*^P < 0.05, ^**^P < 0.01). SF1 and Wnt4 are the downstream transcriptional factors of FSH signaling that regulate the aromatase activity of granulosa cells (Parakh et al., 2006; Pelusi et al., 2008; Boyer et al., 2010). FSH was also found to activate the mammalian target of rapamycin complex 1 (mTOR), which is necessary for the transcription of several proteins related to follicular development (Alam et al., 2004). Collectively, these results suggest that SHP2 plays a dual role in the granulosa and cumulus cells of oocytes. SHP2 interacts with ERα for its transcriptional activity and transduces FSH signaling for meiotic resumption and maturation.

## Discussion

In this study, we identified a novel relationship between SHP2 and ERα in bovine cumulus and human granulosa cells. ERα, a transcriptional regulator of *NPPC* and *NPR2* genes in granulosa cells, interacts with SHP2 for its transcriptional activity. The SHP2/ERα complex is highly expressed in the cumulus cells of immature oocytes (GV stage) but is significantly reduced in the cumulus cells of mature oocytes (MII stage). Human immortalized granulosa cells (COV434) also express the SHP2/ERα complex, which shows a different expression with FSH or E_2_ supplementation. Furthermore, SHP2 knockdown reduced the ERα-transcribed *NPPC* and *NPR2* genes in granulosa cells, but PHPS1-mediated SHP2 inhibition had no significant effect on the SHP2/ERα complex.

Intercommunication between the oocyte and its surrounding granulosa cells is critical for the production of a mature oocyte capable of fertilization, as these cells provide 85% of the nutrients, including growth factors, amino acids, and other energy sources, to the oocyte (Albertini et al., 2001; Eppig, 2001; Sugiura et al., 2005). Granulosa cells also play a vital role in oocyte meiotic arrest and resumption, two critical phenomena that determine the entire reproductive potential of females (Zhang et al., 2010). During the meiotic arrest or pre-ovulatory stage of ovarian follicles, granulosa cells maintain oocyte meiotic arrest by expressing NPPC and its receptor, NPR2. Several studies have recognized that the NPPC/NPR2 system is also essential for the morphological and genetic health of an oocyte (Zhang et al., 2015; Celik et al., 2019). A previous study stated that ERα is the upstream regulator of NPPC/NPR2 governing oocyte meiotic arrest in granulosa cells (Liu et al., 2017). ERα is a ligand-activated transcription factor, and we found that it requires nucleus-localized SHP2 for the regulation of *NPPC* and *NPR2* genes in cumulus and granulosa cells. A previous study detected the ERα and SHP2 complex in breast tissues and confirmed its cytoplasmic localization and involvement in triggering MAP kinases and AKT signaling (Li et al., 2014). In our study, the co-immunoprecipitation analysis revealed that ERα forms a physiological complex with SHP2 in the cumulus cells of GV-stage oocytes, and this complex becomes significantly reduced with the progression of oocyte meiotic maturation *in vitro*. Furthermore, to determine the localization of ERα/SHP2 complex, we found that the cumulus cells of immature (GV) oocytes showed nuclear localization of the ERα/SHP2 complex, but those of mature (MII) oocytes showed cytoplasmic localization of both proteins. The nucleus-localized SHP2's role in ERα transcriptional activity has been previously identified in uterine tissues, but it dephosphorylates ERα and recruits it to the target gene without making a complex (Ran et al., 2017). We found a physiological complex of SHP2/ERα in granulosa cells, and the knockdown of SHP2 highly affected ERα-targeted *NPPC* and *NPR2* genes. Additionally, cultured granulosa cells (COV434) express the SHP2/ERα complex, and the FSH and E_2_ stimulation of granulosa cells has significant effects on this complex.

To analyze the probable structure of the SHP2/ERα complex, we applied a series of computer modeling methods, including protein–protein molecular docking, molecular dynamic simulation, and MM-GBSA calculations, to identify the binding modes of ERα and SHP2. Similar approaches for protein–protein interaction or complex generation have been used in previous studies (Selent et al., 2013; Ge et al., 2018; Wu et al., 2020). Initially, 50 models of the SHP2/ERα complex were generated *via* the online webserver HDOCK. Subsequently, the cluster analysis in Discovery Studio revealed that three different binding modes could be possible for ERα and SHP2. We further applied MD simulations to select the best model for the interaction between SHP2 and ERα. Based on the RMSD values, binding free energies, and key residue interaction numbers, model 1 was selected as the most probable candidate for the SHP2/ERα complex with DNA. In our wet lab data, we found that PHPS1, a site-directed inhibitor of SHP2, has no substantial effects on SHP2 nuclear localization and its involvement with ERα transcriptional activity (Hellmuth et al., 2008). After observing the key residues that are involved in ERα (Leu130, Thr131, His308, Trp484, and Thr688) and SHP2 (Trp248, Gln256, Asn336, Glu379, and Gln506) complex formation, we found that PHPS1 binds to SHP2 in a similar range (Hellmuth et al., 2008). Therefore, PHPS1 is unable to bind with SHP2 and inhibit its nuclear localization in cumulus and granulosa cells.

**Figure 7:**
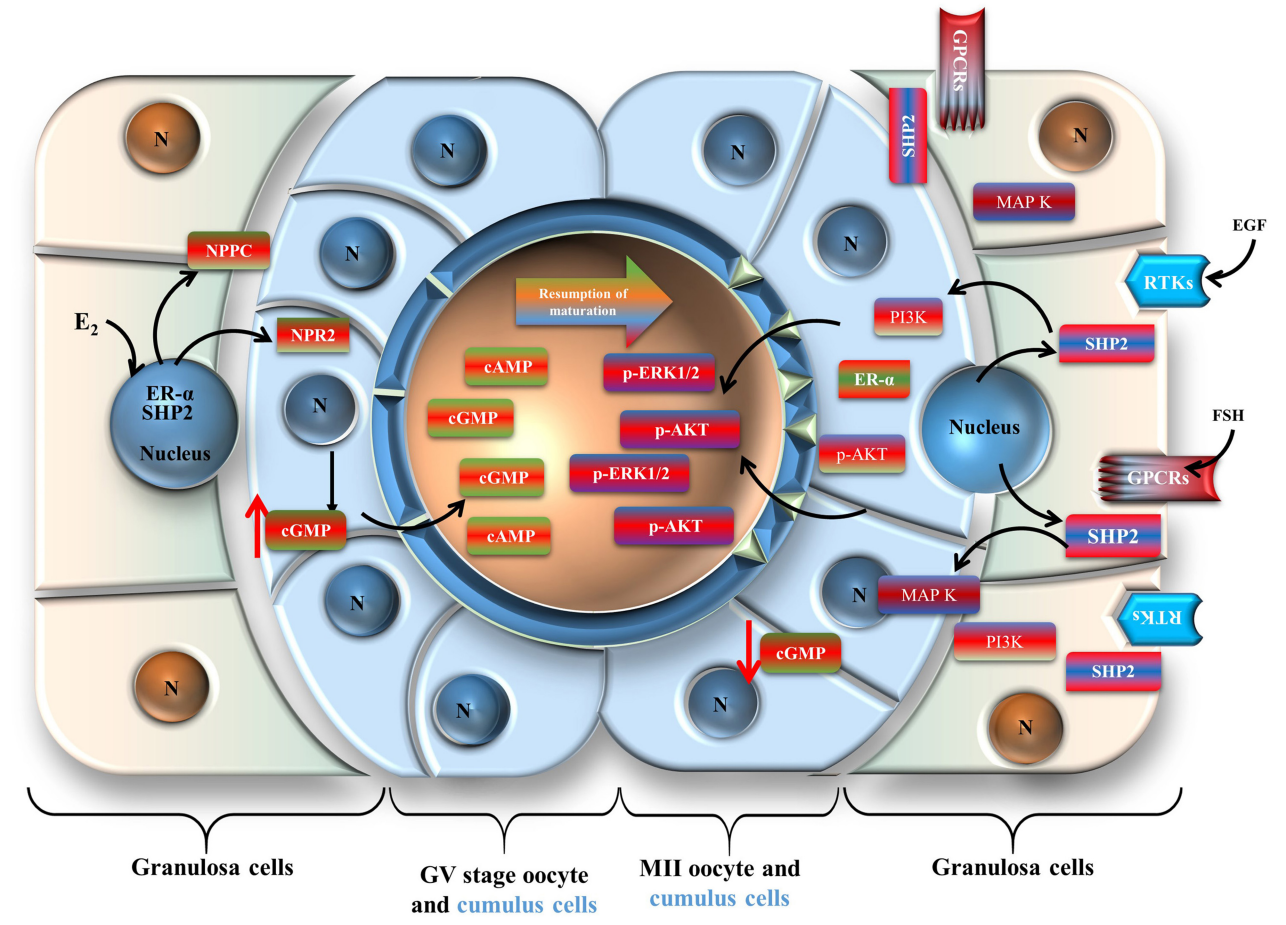
Schematic presentation of the Src-homology-2-containing phosphotyrosine phosphatase regulation of oocyte meiotic maturation. Cumulus cells are shown in light blue, and granulosa cells are shown in reddish green.

SHP2 not only interacts with ERα to regulate meiotic arrest, but it also transduces FSH receptor signaling in granulosa cells. SHP2 is a classic cytoplasmic protein, is a core component of growth factor and cytokine signal transduction, and is essential for oocyte maturation and embryo development (Saxton et al., 1997; Idrees et al., 2019b; Kim et al., 2019). SHP2 is mostly found in two states, an inactive or auto-inhibition state and an active state. During the inactive or auto-inhibition state, the N-terminal SH2 domain blocks the PTP domain, while in the active state, the SH2 domain binds to specific phosphotyrosine sites on the adaptor proteins of receptors (Hof et al., 1998; Neel et al., 2003). Active cytoplasmic SHP2 interacts with the transmembrane adaptor proteins of RTKs and GPCRs and activates MAP kinases, cell cycle controller p34 (CDC2), and PI3K/AKT signaling, which are involved in oocyte GVBD and meiotic progression (Wehrend and Meinecke, 2001; Lin et al., 2009). SHP2 plays a critical role in FSH receptor-induced ERK1/2 and PI3K/AKT signaling in granulosa cells (Donaubauer et al., 2016). In our study, we found significantly enhanced SHP2 cytoplasmic localization during *in vitro* oocyte maturation or with the cells (cumulus or granulosa) exposed to FSH. It may be possible that, during LH surge, SHP2 move toward the cytoplasm and transduce FSH signaling in granulosa cells (Figure 7), but this mechanism of SHP2 needs further verification in *in vivo* animal models. Furthermore, SHP2 knockdown significantly affected FSHR signaling, targeting downstream transcriptional factors such as SF1, Wnt4, and p-mTOR, which regulate the aromatase activity of granulosa cells and play a key role in follicular development (Alam et al., 2004; Parakh et al., 2006; Pelusi et al., 2008; Boyer et al., 2010).

## Conclusion

Taken together, our findings clearly demonstrate that nuclear SHP2 is involved in ERα transcriptional activity for the promotion of *NPPC* and *NPR2* genes related to oocyte meiotic arrest. Furthermore, FSH or growth factors restrict SHP2 interaction with ERα by promoting its export from the nucleus to transduce RTKs or GPCR-dependent signaling in oocyte somatic cells during meiotic resumption. These findings could contribute to opening new avenues of research to understand the process of oocyte meiosis in the mammalian ovary.

## Data Availability Statement

The original contributions generated for the study are included in the article/supplementary material, further inquiries can be directed to the corresponding author/s.

## Ethics Statement

The animal study was reviewed and approved by All experiments, including surgical procedures, were approved by the Gyeongsang National University Institute of Animal Care Committee (GNU-130902-A0059).

## Author Contributions

MI designed the research and analyzed the data. MI and VK performed the research, M-DJ provided reagents and helped in the experiments. MI, VK, and NA wrote the paper. K-WL and I-KK reviewed the paper and supervised the study. All authors contributed to the article and approved the submitted version.

## Conflict of Interest

I-KK was employed by company The King Kong Corp. Ltd. The remaining authors declare that the research was conducted in the absence of any commercial or financial relationships that could be construed as a potential conflict of interest.
