# Supplementary material for: SHP2 Nuclear/Cytoplasmic Trafficking in Granulosa Cells Is Essential for Oocyte Meiotic Resumption and Maturation
Source: Front Cell Dev Biol. 2021 Jan 22;8:611503. doi: 10.3389/fcell.2020.611503 (PMC7862566; doi:10.3389/fcell.2020.611503)
Supplement: Supplementary file 1 [file Data_Sheet_1.docx]

**Supplementary Materials:**

**Table S1.** The calculated docking energy scores (kcal/mol) using HDOCK webserver for top 10 ERα and SHP2 complexes.

| Model | Model1 | Model2 | Model3 | Model4 | Model5 | Model6 | Model7 | Model8 | Model9 | Model10 |
| --- | --- | --- | --- | --- | --- | --- | --- | --- | --- | --- |
| Docking energy | -252.05 | -248.34 | -231.92 | -228.31 | -227.29 | -227.04 | -223.89 | -223.87 | -223.60 | -221.99 |

**Table S2.** The binding free energies (kcal/mol) calculated using MM/GBSA for three ERα and SHP2 models.

| Model | VDW^a^ | ELE^b^ | GB^c^ | SA^d^ | ΔG Total |
| --- | --- | --- | --- | --- | --- |
| Model1 | -262.05 | -176.07 | 356.85 | -33.94 | -115.2 |
| Model2 | -139.99 | -317.11 | 402.44 | -17.80 | -72.47 |
| Model3 | -121.33 | -514.42 | -574.69 | -18.39 | -79.44 |

^a^Van der Waals energy.

^b^ Electrostatic energy.

^c^ Generalized born electrostatic solvation energy

^d^ Surface area.

**Table S3.** Interacting residues of ERα and SHP2 in Model 1.

| Hydrogen Bonds | | | | | Electrostatic Interactions | | Hydrophobic Interactions | |
| --- | --- | --- | --- | --- | --- | --- | --- | --- |
| hERα | | SHP2 | | Distance | hERα | SHP2 | hERα | SHP2 |
| Residues | Atoms | Residues | Atoms | (Å) | Residues | Resides | Residues | Residues |
| Thr48 | OG1 | Gln335 | HE22 | 1.98 | Asp141 | Lys364 | Leu130 | Ala509 |
| Asp50 | OD2 | Asn275 | HD22 | 2.03 | Arg435 | Glu379 | Ile307 | Trp248 |
| Arg53 | HH21 | Asn336 | OD1 | 1.99 | Lys436 | Glu379 | Trp484 | Leu262 |
| Asn124 | O | Arg362 | HH11 | 2.50 | Glu439 | Arg384 | Trp484 | Tyr263 |
| Leu126 | O | Arg362 | HH12 | 1.96 | Trp484 | Arg265 | Ala683 | Lys131 |
| Thr131 | HN | Glu508 | OE2 | 1.46 |  |  | Lys684 | His132 |
| Gln134 | HE21 | His426 | ND1 | 2.44 |  |  |  |  |
| Ser137 | OG | His426 | HE2 | 2.59 |  |  |  |  |
| His308 | HE2 | Gln255 | OE1 | 1.97 |  |  |  |  |
| Gln320 | HE22 | Gln256 | OE1 | 1.97 |  |  |  |  |
| Asn410 | HN | Asn298 | O | 1.84 |  |  |  |  |
| Cys413 | O | Gln335 | HE21 | 1.78 |  |  |  |  |
| Cys413 | O | Asn336 | HD22 | 1.84 |  |  |  |  |
| Asn417 | HD21 | Glu379 | O | 2.63 |  |  |  |  |
| Lys436 | HZ1 | Glu379 | OE1 | 2.77 |  |  |  |  |
| Ser474 | HN | Asp303 | OD2 | 1.73 |  |  |  |  |
| Asp477 | HN | Lys266 | O | 2.11 |  |  |  |  |
| Asp477 | OD1 | Gln271 | HE21 | 1.93 |  |  |  |  |
| Asn482 | OD1 | Gln269 | HE22 | 2.48 |  |  |  |  |
| Trp484 | HE1 | Tyr263 | O | 2.19 |  |  |  |  |
| Gly686 | O | Gln214 | HE21 | 1.86 |  |  |  |  |
| Leu687 | O | Gln214 | HE21 | 2.69 |  |  |  |  |
| Thr688 | HG1 | Glu249 | OE2 | 2.01 |  |  |  |  |
| Gln690 | HE22 | Gln245 | OE1 | 1.87 |  |  |  |  |

**Table S4**. Bovine and human qRT-PCR primers with size and conditions.

|  |  |  |  |  | **Bovine** |  |  |  | |  | |
| --- | --- | --- | --- | --- | --- | --- | --- | --- | --- | --- | --- |
| **Gene name** | |  |  | **Sequence** | | **size** | | | **Accession no.** | |  |
| **NPPC** | | **F** | **5'-TCAGCCTCCTCGCATCT-3'** | | | **101** | | | **NM_174125.2** | |  |
|  |  | **R** | **5′-ACAGCTGGTGTTGTATCC-3'** | | |  |  |  |  |  |  |
| **NPR2** | | **F** | **5′-ATGACAGCATCAACCTGGACTGGA-3′** | | | **145** | | | **NM_174126.2** | |  |
|  |  | **R** | **5′-AGCACGAAACGACTATCCACCACA-3'** | | |  |  |  |  |  |  |
| **PTPN11** | | **F** | **5′-GGCACAGTACTACAACTCAA-3′** | | | **100** | | | **XM_002694590.6** | |  |
|  |  | **R** | **5′-TGGTCTCAGCTAATTTGCTT-3'** | | |  |  |  |  |  |  |
| **MAP K1** | | **F** | **5′-CCGTGTTGCAGATCCAGAC-3′** | | | **130** | | | **NM_175793** | |  |
|  |  | **R** | **5′-GACGGACCAGATGTCGATG-3'** | | |  |  |  |  |  |  |
| **AKT3** | | **F** | **5′-AGCTGTTTTTCCATTTGTCG-3′** | | | **94** | | | **NM_001191309.1** | |  |
|  |  | **R** | **5′-TGTAGATAGTCCAAGGCAGA-3'** | | |  |  |  |  |  |  |
| **CX43** | | **F** | **5′-TGGCATTGAAGAGCACGGCA-3′** | | | **104 NM_174068.2** | | | | |  |
|  |  | **R** | **5′-TCAGCAAGAAGGCCACCTCGA-3′** | | |  |  |  |  |  |  |
| **CX37** | | **F** | **5′ -AGCCCGTGTTTGTGTGCCAG-3′** | | | **121 NM_001083738.1** | | | | |  |
|  |  | **R** | **5′-ACCAGGGAGATGAGTCCGACCA-3′** | | |  |  |  |  |  |  |
| **GAPDH** | | **F** | **5′-CCCAGAATATCATCCCTGCT-3′** | | | **185 NM_001034034** | | | | |  |
|  |  | **R** | **5′-CTGCTTCACCACCTTCTTGA-3′** | | |  |  |  |  |  |  |
|  |  |  |  |  | **Human** |  |  |  | |  |  |
| **NPPC** | | **F** | **5′-CGCAAATACAAAGGAGCCAACAAG-3′** | | | **165** | | | **NM_024409.4** | |  |
|  |  | **R** | **5-AGGCTGATGACCAAAGATGACCTC-3′** | | |  |  |  |  |  |  |
| **NPR2** | | **F** | **5′-TGACCGGACCCAACTGAATG-3′** | | | **171** | | | **NM_003995.4** | |  |
|  |  | **R** | **5′-GCATACTGTTCCATGCGCAG-3′** | | |  |  |  |  |  |  |
| **GAPDH** | | **F** | **5′-CAATGCCTCCTGCACCACCA-3′** | | | **175 NM_002046** | | | | |  |
|  |  | **R** | **5′-GATGTTCTGGAGAGCCCCGC-3′** | | |  |  |  |  |  |  |


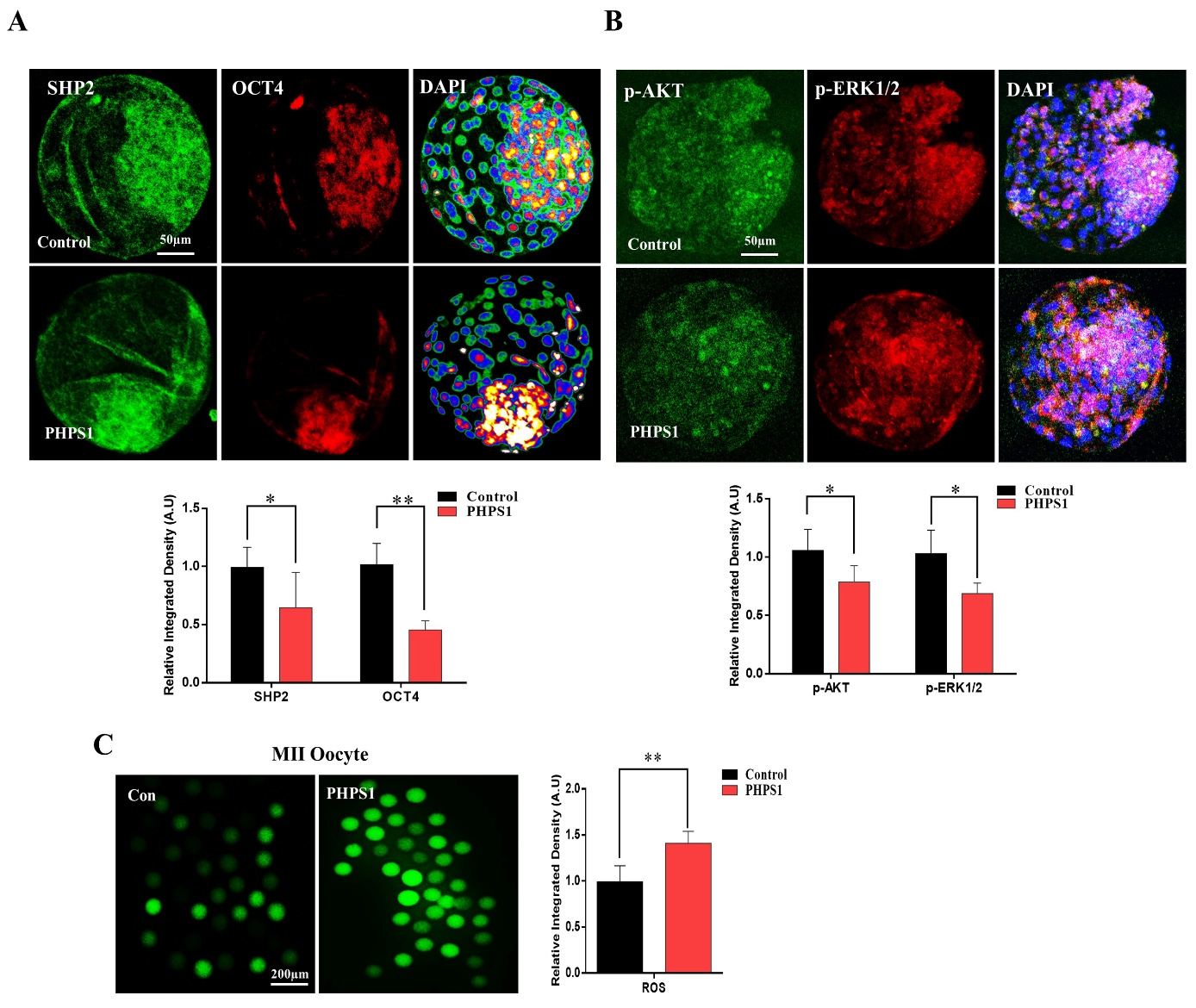


**Figure S1**. (**A**) Sarcoma-homology-2-containing phosphotyrosine phosphatase (SHP2) and OCT-4 immunofluorescent expressions analyzed in control and phenyl hydrazonopyrazolone sulfonate 1 (PHPS)-treated Day 8 bovine blastocysts. (**B**) SHP2 downstream proteins like p-AKT and p-ERK1/2 analyzed via immunofluorescence in Day 8 bovine blastocyst.(**C**) Qualitative analysis of the reactive oxygen species (ROS, H_2_DCFDA) in MII-stage oocytes. H_2_DCFDA staining was used to detect the level of ROS in the control and PHPS1-treated oocytes. Bar = 200 µm.


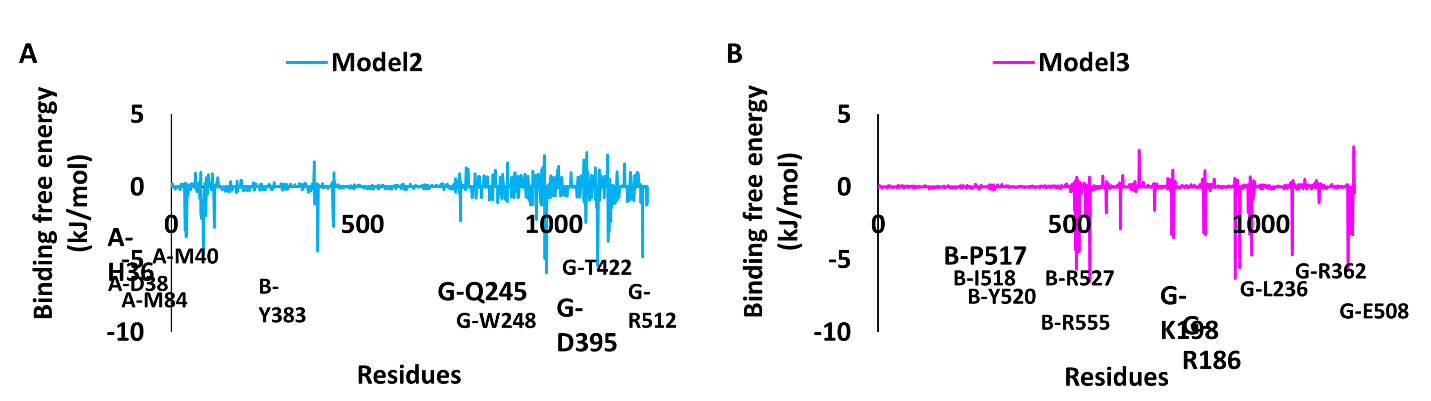


**Figure S2**. Decomposition of the MM-GBSA energy for each amino acid of the binding surface from ERα and SHP2. Key residues from both proteins involved in interactions are highlighted A) Model2, B) Model3.


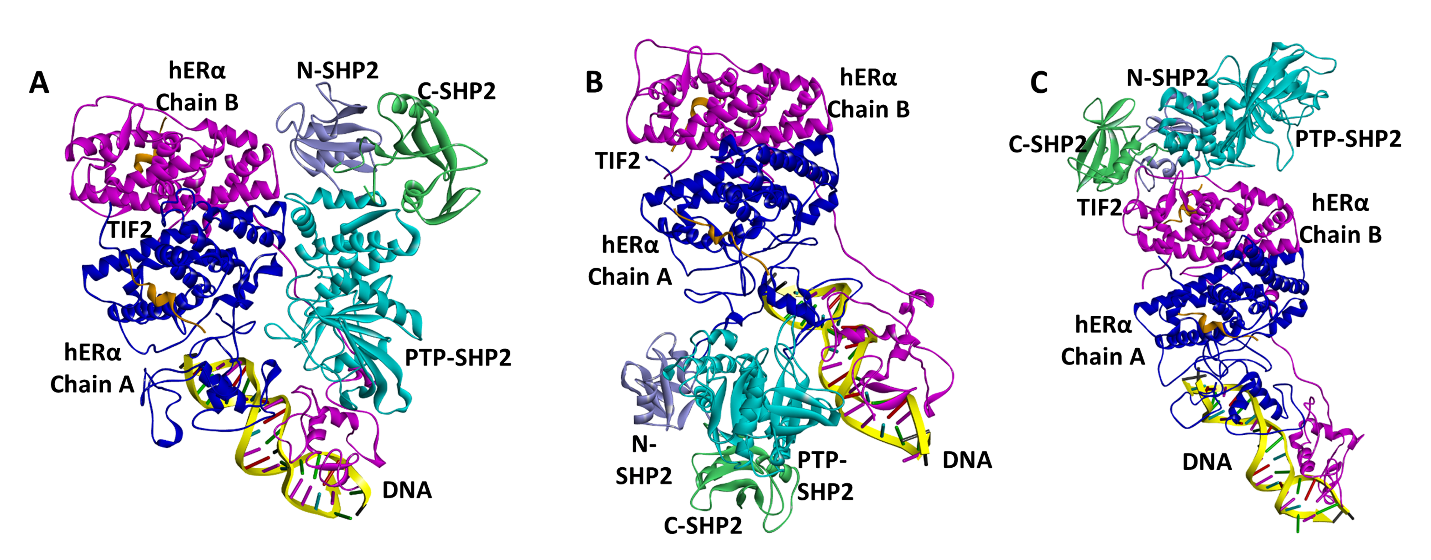


**Figure S3.** Top three ranked models binding mode A) Model 1, B) Model 2, C) Model 3. ERα protein domains are shown in pink (chain B), blue (chain A), orange (TIF2), yellow (DNA) colors, whereas SHP2 protein domains are shown in green (C-SHP2), purple (N-SHP2) and cyan (PTP) respectively.
